# Systems and Circuits Linking Chronic Pain and Circadian Rhythms

**DOI:** 10.3389/fnins.2021.705173

**Published:** 2021-07-02

**Authors:** Andrew E. Warfield, Jonathan F. Prather, William D. Todd

**Affiliations:** Program in Neuroscience, Department of Zoology and Physiology, University of Wyoming, Laramie, WY, United States

**Keywords:** chronic pain, circadian rhythms, suprachiasmatic nucleus, lateral parabrachial nucleus, subparaventricular zone, inflammatory pain, neuropathic pain, immune system

## Abstract

Research over the last 20 years regarding the link between circadian rhythms and chronic pain pathology has suggested interconnected mechanisms that are not fully understood. Strong evidence for a bidirectional relationship between circadian function and pain has been revealed through inflammatory and immune studies as well as neuropathic ones. However, one limitation of many of these studies is a focus on only a few molecules or cell types, often within only one region of the brain or spinal cord, rather than systems-level interactions. To address this, our review will examine the circadian system as a whole, from the intracellular genetic machinery that controls its timing mechanism to its input and output circuits, and how chronic pain, whether inflammatory or neuropathic, may mediate or be driven by changes in these processes. We will investigate how rhythms of circadian clock gene expression and behavior, immune cells, cytokines, chemokines, intracellular signaling, and glial cells affect and are affected by chronic pain in animal models and human pathologies. We will also discuss key areas in both circadian rhythms and chronic pain that are sexually dimorphic. Understanding the overlapping mechanisms and complex interplay between pain and circadian mediators, the various nuclei they affect, and how they differ between sexes, will be crucial to move forward in developing treatments for chronic pain and for determining how and when they will achieve their maximum efficacy.

## Introduction

Research focused on the intersection between circadian rhythms and pain has revealed numerous bidirectional interactions among the processes underlying both systems. Such interactions have included the timing of pain perception and inflammatory responses, the impact of disrupted circadian clock gene expression following injury and its role in the induction and maintenance of chronic pain, the influence of chronic pain on peripheral and central circadian rhythms, and temporal differences in the efficacy of pain-related interventions. Understanding the mechanisms underlying these interactions and how to implement treatments based on these findings will be vital to the better management of chronic pain. In the United States, estimates of the prevalence of chronic pain are as high as 40% of the population, or roughly 100 million people (Tsang et al., 2008). The opioid epidemic has drastically limited the options to treat pain for healthcare workers (Volkow and Collins, 2017; Price et al., 2018), not to mention that opioids often exhibit inadequate efficacy (Johannes et al., 2010), and can lead to tolerance and addiction (Damiescu et al., 2021). Altogether, this represents a public health crisis that is not being effectively handled.

Furthermore, drugs like morphine, gabapentin and the tricyclic nortriptyline do not change the rhythmicity of chronic pain episodes (Odrcich et al., 2006; Gilron et al., 2013). In these studies, neuropathic pain was found to worsen throughout the day regardless of these traditional analgesics and this day–night difference was worse in females than in males. Additionally, morphine, which binds to mu opioid receptors, is significantly more effective in men than women (Doyle et al., 2017; Doyle and Murphy, 2018). Gabapentin blocks the α2δ-1 subunit of voltage gated calcium channels (Gee et al., 1996), which show a 24-h rhythm of protein level that determines when this drug will be most effective at relieving allodynia in mice (Kusunose et al., 2010). Additionally, the α2δ-1 subunit binding to its endogenous ligand mediates neuropathic pain through excitatory synaptogenesis (Park et al., 2018; Yu et al., 2018), which implies a role for gabapentin in limiting a gain in excitability in neurons of pain networks if administered at the correct chronobiological time point. Rhythms of pain and inflammation, as well as rhythms of their mediators, are common in patients with cancer (Saini et al., 2013), HIV with neuropathic pain (Shi et al., 2012), postherpetic neuralgia and diabetic peripheral neuropathy (Gilron et al., 2013), fibromyalgia (Torpy et al., 2000), multiple inflammatory and autoimmune conditions (Smolensky et al., 2007; Buttgereit et al., 2015; Comas et al., 2017), and neuropathic conditions (Celik et al., 2012; Crodelle et al., 2019). Additionally, inflammatory pain is most frequently reported to be worst in the morning (Buttgereit et al., 2015), while neuropathic pain is most frequently reported to be worst at night (Odrcich et al., 2006; Celik et al., 2012). Translating the mechanisms underlying these rhythms into a clinical benefit presents an opportunity to better care for chronic pain patients.

Interestingly, chronotherapy (the practice of administering treatments at certain time points or directly influencing the circadian system guided by circadian physiology rather than at the convenience of patients or practitioners) has been shown to have a beneficial clinical impact. Taking immunosuppressants, like glucocorticoids, at night instead of the morning has been shown to improve the morning symptoms of rheumatoid arthritis (RA), including pain (Buttgereit et al., 2015; Walton et al., 2020). Additionally, bright light therapy (BLT), which is known to act upon the circadian system (Rubino et al., 2020), has been shown to reduce self-reported pain scores in humans even when done only once a week (Leichtfried et al., 2014). This study had participants come in at a consistent time between 7:30 am and 1:30 pm once a week for 3 weeks. Similar work is warranted to test the efficacy of more frequent BLT and to measure differences of BLT administered at various circadian time points. In rats, both green and blue LED light exposure for 8-h periods over 5 days was shown to induce analgesia, and green light decreased both thermal and neuropathic pain (Ibrahim et al., 2017). Specialized glasses that filtered out non-green light were also effective, providing further clinical relevance to these findings. Importantly, this reduction in both hyperalgesia and mechanical allodynia was maintained 4 days after the light exposure had ended. Importantly, the circadian system responds to both blue and green light (Lockley et al., 2003), although it induces stronger phase shifts to blue light. The melanopsin cells that mediate these light-induced circadian changes also project to other areas of the brain including the thalamus (Noseda et al., 2010), where pain signals and other sensory information are relayed on to their relevant regions of the cerebral cortex. Intrathecal administration of naloxone, a competitive inhibitor of opioid receptors, or lidocaine, a sodium channel blocker, both inhibit the analgesic effect caused by green light, implying such stimulation leads to analgesia through an endogenous opioid pathway (Ibrahim et al., 2017). Indeed, endogenous opioids and their receptors exhibit circadian rhythms as well (Oliverio et al., 1982; Sandoval et al., 2018). Importantly, this implies that the circadian system sets the rhythm of endogenous analgesia and can be driven in a therapeutic fashion.

Notably, such green light therapy showed efficacy in both male and female mice (Ibrahim et al., 2017). However, most chronic pain patients are women while the vast majority of preclinical studies have been done on male rodents (Mogil, 2012). Both circadian rhythms and pain regulation have marked sex differences that must be accounted for when interpreting research seeking to understand the relationship between these two systems. Broadly, female mice seem to be less reliant on microglia for pain signaling than males (Sorge et al., 2015). Female humans seem to have earlier (Duffy et al., 2011) and more variable activity onset compared to males, and female mice have also been found to show larger circadian phase shifts in response to light (Kuljis et al., 2013). In most studies examining chronic pain mechanisms, the expected results occurred in males, but not in females 74.6% of the time (Mogil, 2020), which implies a major roadblock to treating pain in females. In order to avoid this imbalance in the future, sex differences in both the circadian system and in pain must be more thoroughly addressed.

Furthermore, the circadian system itself must become more widely recognized as being influential on numerous processes, including sleep. Pain has been characterized as among the internal causes of circadian disruption in critically ill patients (Oldham et al., 2016), and in fact, pain is a comorbidity with numerous conditions, especially sleep disturbances. Both chronic opioid use and chronic pain lead to sleep disturbances including sleep apnea (Onen et al., 2005; Shaw et al., 2005; Webster et al., 2008). Importantly, the interaction between sleep and chronic pain seems to be bidirectional (Raymond et al., 2001; Benca et al., 2004; Taylor et al., 2007). Acute sleep deprivation has been implicated as a modulator of pain in the following day (Lautenbacher et al., 2006; Roehrs et al., 2006; Alexandre et al., 2017), and distraction analgesia and secondary hyperalgesia are decreased and increased, respectively, in people who had slept less than 6.5 h a night during the month before (Campbell et al., 2011). A large telephone study showed that sleep duration can predict next day pain in humans (Edwards et al., 2008), noting that both less than six and more than 9 h of sleep were associated with increased pain the following day. It is also crucial to reiterate that sleep is under the control of interacting homeostatic and circadian processes (Fuller et al., 2006; Beersma and Gordijn, 2007; Maywood et al., 2021). In patients with sleep disorders, internal rhythms and external zeitgebers are often not aligned, which is a major contribution to the observed sleep disturbances (Chowdhury et al., 2019). This implies that disruption of the underlying circadian regulation of sleep may also be a major driver of pain, rather than simply the amount of sleep.

The circadian system also appears to directly regulate perceptual processing. In mice, it has been shown that the 50% paw withdrawal threshold to a painful stimulus increases throughout the light period with a peak at zeitgeber time 15/19 (ZT15/19, ZT0 = lights on), when measured every 4 h (Minett et al., 2014). Importantly, mice without sodium voltage gated channel 1.8 (NaV1.8), which mediates nociception, showed the same rhythm in response to light touch. This implies a rhythm in mechanosensory sensitivity that is most likely a product of the central nervous system and its circadian timing system. Recently, a model describing the rhythmicity of pain sensitivity in humans to four different types of acute pain, including heat and electrical stimulation pain, has been described and been shown to be predictable based on a mathematical model (Crodelle et al., 2019). This pain rhythm correlates to a decrease in A-beta fiber input and an increase in c-fiber input and was measured in relation to time since waking of the patients in each study. The sleep-wake cycle effects on this rhythm cannot be separated from that of the endogenous rhythm, however, the recognition of rhythmicity in various types of acute pain cannot be denied. Additionally, this rhythm levels out and decreases toward the end of the 24-h period following wake, which argues for a rhythm that is not entirely dependent on the homeostatic influence of sleep. In the coming years, it will be crucial to take the existing knowledge regarding the mechanisms of pain and circadian rhythms to apply the overlapping mediators and circuits to treatment plans for patients that are suffering from dysfunction of both systems. In this review we will highlight our current understanding of the link between circadian rhythms and chronic pain, and we will argue for a larger scope of focus in research on pain and circadian rhythms that includes a systems-level and circuit-based approach.

## Circadian Rhythmicity

Nearly all organisms have endogenously generated rhythms that follow an approximately 24-h pattern, which persist under constant conditions and are termed “circadian,” and that can be synchronized to the environmental light-dark cycle (Patke et al., 2020). These rhythms are mediated at the molecular level by a transcription-translation feedback loop (TTFL), the mechanisms of which are mostly conserved among all mammalian cells (Andreani et al., 2015). Strikingly, almost half of the known mammalian proteins show circadian regulation of their transcription (Zhang et al., 2014). The TTFL consists of the positive regulators BMAL1 and CLOCK which, beginning at circadian dawn, heterodimerize and bind to their E-box promoter to drive the expression of the negative regulators PER1/2/3 and CRY1/2. These negative regulators heterodimerize and build up in the cytoplasm, and by circadian dusk such PER-CRY complexes relocate to the nucleus to inhibit BMAL1-CLOCK, thus inhibiting their own transcription. Over the course of the night the PER-CRY complexes degrade, completing the TTFL which takes about 24 h in total (Lowrey and Takahashi, 2004). BMAL1-CLOCK also similarly drives the expression of other regulators that form accessory loops to stabilize the TTFL. REV-ERBα/β are negative regulators of BMAL1 and as such, binding to their E-box leads to less BMAL1 protein being made, whereas ROR’s are positive regulators of BMAL1 synthesis and therefore lead to more BMAL1 protein (Ko and Takahashi, 2006; Cho et al., 2012; Kojetin and Burris, 2014; Zhang et al., 2014).

The suprachiasmatic nucleus (SCN) of the hypothalamus is the master pacemaker of the mammalian circadian system and is required for synchronizing TTFLs in cells throughout the brain and body. SCN neurons receive innervation from the retinohypothalamic tract (RHT) and entrain their rhythms to light via this connection (Hastings et al., 2018). The extracellular signal-related kinase (ERK) signaling pathway appears to mediate the connection between photic input and clock entrainment to light in SCN cells (Butcher et al., 2002). In fact, light stimulation during the dark phase phosphorylates both ERK and CREB (cAMP Response element binding protein) in the same cells, and glutamate stimulation, which is one of the neurotransmitters released from the RHT onto SCN neurons, leads to the same response. Phosphorylated ERK (pERK) shows a rhythm that increases until late in the light phase and then decreases until late in the dark phase before rising again (Obrietan et al., 1998; Cao et al., 2011). The levels of PER proteins also rise throughout the day before relocating to the nucleus to inhibit their own transcription (Brancaccio et al., 2019), and pERK has been shown to stimulate *Per1* expression in a CREB dependent manner (Travnickova-Bendova et al., 2002). pERK has also been shown to lead to transcription of immediate early genes associated with cellular activation such as c-fos (Dziema et al., 2003). In addition, pERK mediates translational activation via an mTOR pathway, which includes decreasing the translation of vasoactive intestinal peptide (VIP) mRNA in SCN neurons and increasing PER and CRY proteins throughout the light period (Cao et al., 2013). The regulation of PER, CRY, and VIP proteins implicate pERK as a crucial regulator of both entrainment of internal rhythms to external zeitgebers, the dysregulation of which underlies sleep disorders as mentioned above, and of the core negative feedback loop within the TTFL of the SCN. Importantly, and as we will elaborate upon below, pERK regulates transcription, translation and post-translational modifications of many of the central neuronal mediators of chronic pain (Latremoliere and Woolf, 2009). The role of pERK in decreasing VIP translation while increasing the expression of clock gene proteins raises the intriguing possibility that increased pERK in the SCN from sources other than light, such as pain, could cause desynchrony among SCN cells. Beyond this, pain may be able to compete with light as a zeitgeber in such cells, given the transient rise in pERK seen in animal models of pain and temporal nature of pain episodes seen in patients with chronic pain pathologies.

Indeed, a subpopulation of SCN neurons expressing VIP appears to be the main synchronizers of behavioral rhythms (Todd et al., 2020), and VIP signaling and its receptor VPAC2 have been shown to be essential for such synchrony (Hamnett et al., 2019). Interestingly, VIP signaling onto other cells in the SCN relies upon ERK phosphorylation but does not involve CREB, whereas light entrainment of the SCN through glutamate signaling onto VIP cells relies on both ERK and CREB (Hamnett et al., 2019), and pERK inhibition significantly reduces the amplitude of VIP-induced luciferase rhythms in the SCN. Additionally, phosphorylated c-jun N-terminal kinase (pJNK) and brain-derived neurotrophic factor (BDNF) seem to play roles in mediating period length and phase shifts, respectively, via VIP signaling (Liang et al., 2000; Hamnett et al., 2019). Importantly, both JNK and BDNF also play roles in inflammation and pain, which will be discussed in more detail below. Sex also plays a role in VIP signaling as male mice have more VIP-positive neurons in the SCN and the acrophase of VIP expression in males has been reported to be as much as 8 h later than in females (Bailey and Silver, 2014). VIP neurons have been shown to be composed of two distinct subgroups (Todd et al., 2020), one of which expresses gastrin releasing peptide (GRP) and is thought to be the site of non-photic input, including androgen action onto SCN androgen receptors that are also expressed more heavily in the male SCN than in females. The other VIP population, expressing neuromedin S (NMS), appear to function as the main pace-making cells of the SCN (Todd et al., 2020).

The SCN coordinates numerous circadian behavioral and physiological rhythms including locomotor activity (LMA), wake-sleep, feeding, aggression, body temperature, and melatonin and corticosteroid secretion (Saper, 2013; Todd et al., 2018; Todd et al., 2020). The SCN accomplishes such synchrony primarily through multiple neural circuits leading to various nuclei within the brain (Saper, 2013). The major output pathway of the SCN runs through the subparaventricular zone (SPZ), an adjacent area just dorsal to it and extending slightly caudally (Abrahamson and Moore, 2001; Leak and Moore, 2001; Lu et al., 2001). The SPZ appears to be a heterogeneous structure comprised of subregions with distinct projection patterns that regulate particular circadian rhythms, with a ventral portion that mediates rhythms of LMA and wake-sleep through its projection to the dorsomedial hypothalamus (DMH) (Lu et al., 2001; Chou et al., 2003; Saper, 2013), and a dorsal portion that mediates rhythms in aggression propensity through its projection to the ventromedial hypothalamus (VMH) (Todd et al., 2018). Crucially, the SPZ has also recently been shown to send projections to the lateral parabrachial (LPB) nucleus in the midbrain (Vujovic et al., 2015). The LPB has been extensively shown to play a vital role in the relay of pain-related information from the spinal cord to the forebrain, and through these connections the LPB appears to mediate emotional and learning-based aspects of pain (Chiang et al., 2020; Sun et al., 2020). As we will elaborate on in this review, the SCN → SPZ → LPB pathway may serve as a circuit substrate through which the circadian system could exert its influence and lead to the reported temporal differences in pain perception and sensitivity across the 24h day.

Additionally, a recent study that utilized highly specific Cre-dependent retrograde tracing via a pseudorabies virus identified several inputs to SCN^VIP^ neurons that included the LPB (Todd et al., 2020). Moreover, anatomical drawings of Cre-dependent anterograde tracing from glutamatergic cells and from Foxp2- and pro-dynorphin (pdyn)-expressing cells, two subsets of these glutamatergic neurons, within the LPB also revealed dense fibers in the region of the hypothalamus corresponding to the SPZ (Huang et al., 2021). The presence of such LPB → SCN^VIP^ and LPB → SPZ pathways raises the intriguing possibility of circuits whereby chronic pain could underlie circadian disruption, an understudied topic that warrants further investigation. Indeed, inputs to the circadian system other than the RHT are already well known to play important roles in modulating circadian function. The intergeniculate leaflet (IGL) also sends a substantial input to SCN^VIP^ neurons (Todd et al., 2020), via the geniculohypothalamic tract (GHT), and has been implicated in mediating non-photic entrainment via the release of neuropeptide Y (NPY) onto SCN cells (Biello et al., 1994; Janik and Mrosovsky, 1994; Janik et al., 1994). Interestingly, NPY has also been shown to be a key element in modulating neuropathic and inflammatory pain, perhaps through its inhibitory actions on microglia (Carniglia et al., 2017), which as we will elaborate upon below, play their own critical role in pain signaling. Taken together, these findings underscore the importance of analyzing the interaction between circadian rhythms and pain from the perspective of circuits and systems.

## Peripheral Activation

Several different types of immune cells mediate the peripheral release of cytokines throughout the body and the vast majority of such cells are under circadian control. Circadian rhythms have been shown in peripheral macrophages and monocytes (Boivin et al., 2003; Hayashi et al., 2007; Keller et al., 2009), T cells (Bollinger et al., 2011), NK cells (Arjona and Sarkar, 2006), and dendritic cells and B cells (Silver et al., 2012). Chemokines and cytokines are also released and expressed under the control of circadian clock genes. Monocytes release CCL2/8 under the influence of BMAL1 and CLOCK (Garaulet et al., 2010; Nguyen et al., 2013), and express CCR2 under the influence of PER1 (Wang et al., 2016). T cells express CCR7 under the influence of BMAL1 (Druzd et al., 2017). NK cells release IL-1β, IL-6 and others under the control of *Per* genes (Liu et al., 2006; Guess et al., 2009; Logan et al., 2013). Macrophages play a large role in peripheral activation by releasing IL-6, CXCL1, and TNF-α under the control of CRY genes (Narasimamurthy et al., 2012; Nakazato et al., 2017), and CCL2/8 under the control of BMAL1 and REV-ERBα (Nguyen et al., 2013; Sato et al., 2014). REV-ERBα also mediates IL-6 expression in macrophages (Gibbs et al., 2012). Fibroblasts, epithelial cells, and mast cells also play a role through expression of IL-6 and TNF-α mediated by *Cry* genes (Nakazato et al., 2017), CXCL5 mediated by BMAL1 (Gibbs et al., 2014), and IL-6/13 and TNF-α mediated by CLOCK (Kawauchi et al., 2017), respectively. Several reviews of these mediators and their connection to peripheral inflammation and circadian rhythms further elucidate these interactions (Scheiermann et al., 2013; Labrecque and Cermakian, 2015; Woller et al., 2017; Segal et al., 2018; Jain et al., 2020).

Notably, pain in women seems to be more sensitive to such peripheral immune activation as injected lipopolysaccharide (LPS) does not exacerbate pain in the face of a noxious stimulus in men but does in women (Karshikoff et al., 2015). It was also shown that systemic LPS impaired conditioned pain modulation in women only. Furthermore, the cytokine response to LPS is greater in women as compared to men (Karshikoff et al., 2015; Doyle and Murphy, 2017). Interestingly, it has been well established that women have shorter circadian periods and earlier chronotypes than men, indicating the internal clock runs faster in females compared to males (Duffy et al., 2011). It is unclear whether these sex differences in circadian function may be related to the sex differences seen in pain-related immune activation. However, given the ubiquitous nature of circadian control among peripheral immune cells, such a link warrants further investigation.

## Central Sensitization

### Gain in Excitation

**FIGURE 1:**
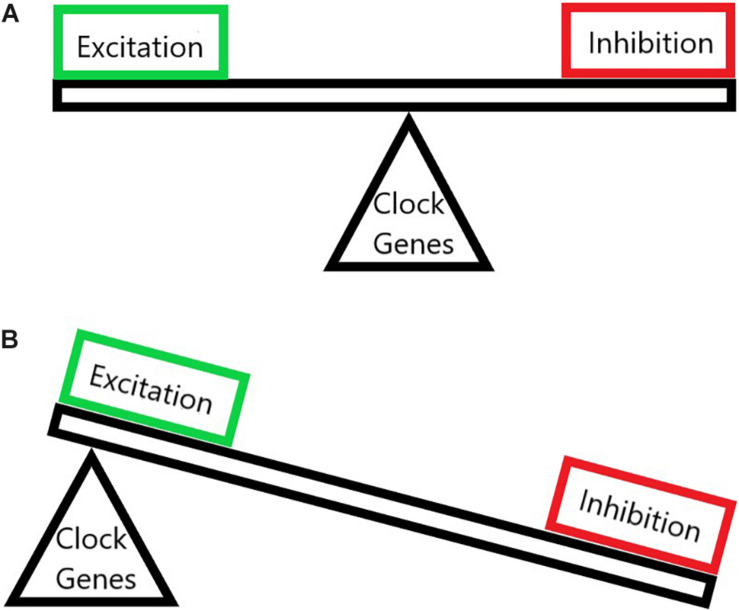
The imbalance of neuronal excitation and inhibition in chronic pain. **(A)** A normal state where the excitation and the inhibition are balanced upon proper circadian clock gene function. **(B)** A chronic pain state where clock genes have been altered thus throwing off the balance of pain pathways and allowing for pathologically increased excitation and decreased inhibition.

The first discovery of a central modulation in post-injury pain came in 1983 when Clifford Woolf showed that, after activation of c-fibers by noxious heat stimulation, the spinal cord of rats exhibited increased excitability and an enlarged receptive field (Woolf, 1983). Since then the mechanisms of this process, now known as central sensitization, have been extensively investigated. Spinal neurons that exhibit central sensitization show increased excitation as well as disinhibition (Figure 1) as well as an expanded receptive field (Latremoliere and Woolf, 2009). Purely activity-driven sensitization is called “wind-up,” and is mediated mainly by substance P, CGRP, and BDNF leading to summation of c-fiber potentials (Sivilotti et al., 1993; Latremoliere, 2016). Substance P produces long-term membrane depolarization upon binding to its receptor NK-1 (Henry, 1976), which is expressed widely throughout spinal projection neurons in lamina 1 (Todd, 2010). Additionally, most pain afferents that contact projection neurons express substance P (Todd, 2010; Peirs et al., 2020), and ablation of NK1R neurons in lamina 1 attenuates responses to highly noxious stimuli (Mantyh et al., 1997). CGRP and BDNF, during wind-up, promote temporal summation of c-fiber inputs (Balkowiec and Katz, 2000; Bird et al., 2006). Together this leads to enough depolarization to remove the magnesium blockade on NMDA receptors. However, this purely activity-based sensitization can be resolved and is therefore not sufficient for lingering chronic pain pathologies (Latremoliere and Woolf, 2009).

Central sensitization, and its gain in excitation, can be sustained through receptor alterations and transcriptional alterations (Woolf and Salter, 2000). There are three main receptor types for glutamate: AMPA (α-amino-3-hydroxy-5-methyl-4-isoxazolepropionic acid), mGluR (metabotropic glutamate receptor), and NMDA (*N*-methyl D-Aspartate). The GluR1/3/4 subunits of AMPA are calcium permeable and Glu2 is not (Polgar et al., 2008), mGluR1/5 metabotropic subunits increase cystolic calcium (Valerio et al., 1997), and the NR2/3 subunits of NMDA are calcium permeable while the NR1 subunit is glycine receptive (Stephenson et al., 2008). Typically NMDA receptors are made of two NR1 subunits and two NR2/NR3 subunits) (Stephenson et al., 2008). Calcium influx is instrumental in initiating and maintaining central sensitization (Latremoliere and Woolf, 2009). In the dorsal horn, mGluR5 and NR2B are concentrated in lamina I/II (Alvarez et al., 2000; Nagy et al., 2004). In addition, neurons in lamina 1 that lack NK1 receptors are heavily packed with GluR4 AMPA receptors (Polgar et al., 2008). This seems to implicate the superficial lamina of the dorsal horn as an easy target for a gain in excitation. Indeed, when rats are treated with complete Freund’s adjuvant (CFA) injected into the intraplantar space, the concentration of mGluR5 increases in these lamina (Pitcher et al., 2007). Under capsaicin pain it has been shown that blocking NMDA receptors, antagonizing mGluR1/5, or giving an agonist for mGluR2/3 will all attenuate mechanical hypersensitivity in rodents (Soliman et al., 2005).

**FIGURE 2:**
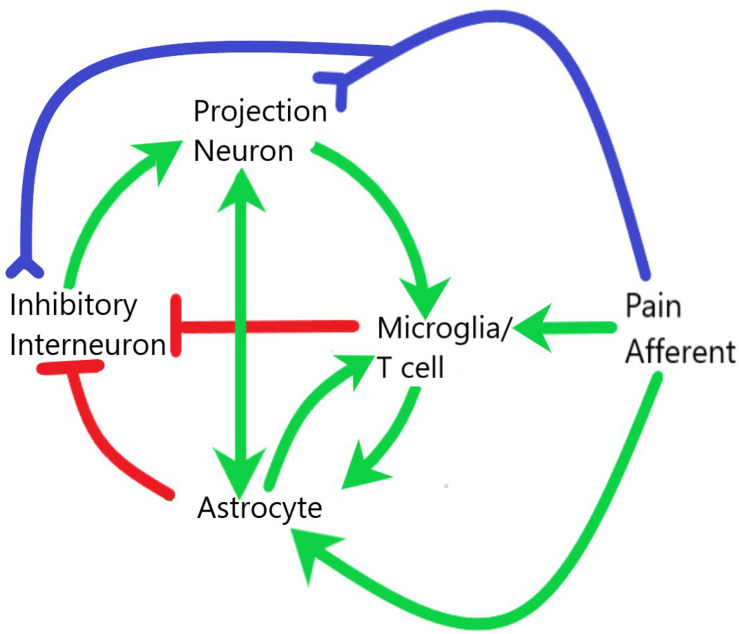
Interaction of glial and neuronal cells within the spinal cord during central sensitization and neuroinflammation. Pain afferents are subject to neuropathic or inflammatory insult and signals of real or potential tissue damage are sent to both the projection neurons and inhibitory interneurons within the dorsal horn of the spinal cord. The release of neuropeptides in the synaptic cleft as well as the increased electrical activity at these synapses leads to activation of both microglia and astrocytes. These glial cells then interact with each other and with neurons to establish a systemic positive feedback system that is self-sustaining. Green lines demarcate activation, red lines demarcate inhibition, blue lines demarcate axonal connection.

LTP can be a heterosynaptic process that is largely dependent on NMDA receptors but also involves AMPA (Ji et al., 2003). TNF-α can induce LTP after nerve injury but this is abolished without the TNF-α receptor (Liu et al., 2007; Park et al., 2011). Interestingly, the TNF-α driven LTP could be blocked by inhibiting pJNK, p-p38, or NF-κB but tetanic stimulation of pain afferents could not be blocked through this mechanism (Liu et al., 2007). This implies a difference between neuroinflammatory (Figure 2 and discussed below) and wind-up-driven central sensitization. Peripheral inflammation triggers a switch from GluR2/3 to GluR1 AMPA subunits, as well as clustering mGluR1/5 at the synapse. The NR2B subunit of NMDA receptors is also phosphorylated (Latremoliere and Woolf, 2009), and this mediates NMDA receptors being sent to the synapse (Mi et al., 2004). All these changes increase activity of NMDA receptors and increase calcium permeability of the cell. Unilateral injection of formalin increases expression of NR2A/B but not glycine binding NR1 in the rostral anterior cingulate cortex (rACC) bilaterally and increased NMDA evoked currents in rACC neurons. Additionally, blockade of NR2A or B in the rACC abolished conditional place aversion and c-fos expression in the rACC (Li et al., 2009). Furthermore, mice in a chronic migraine model had increased phosphorylated NR2B in the trigeminal nucleus caudalis and the inhibition of this relieved allodynia (Wang X.Y. et al., 2018). Importantly, Xia et al. (2016) showed that pain in a chronic constriction model peaks at ZT4 (ZT0 = lights on) and troughs at ZT16 for both hyperalgesia and allodynia. This was found to coincide with the increased levels of pNR2B and NR2B mRNA in the spinal cord and the SCN, and also coincides with the late active period acrophase of human neuropathic pain. This shows both rhythms of expression for key pieces in central sensitization, and that there are systemic issues that influence the master pacemaker for circadian rhythms. However, these rhythms were not evaluated in 24 h darkness conditions so it is possible that they are light dependent instead of truly endogenous. Additionally, in a separate study, NR2B mRNA expression did not exhibit a circadian rhythm when it was analyzed in cell cultures from the dorsal horn (Zhang J. et al., 2012). This implies circadian rhythmicity of NR2B mRNA expression in dorsal horn neurons when they are functionally connected to the central circadian system, but a loss of such rhythms when they are not connected.

In addition to localization of subunits, painful stimulation leads to intracellular changes and signaling cascades that can cause increased abundance and activity of such subunits. One of the most well studied molecules involved in this is ERK, which can be induced in minutes following a painful stimulus (Ji et al., 1999). In addition to playing a role in circadian photoentrainment, as described above, ERK is also induced in neurons following spared nerve injury (Liu et al., 2018), formalin injection (Pezet et al., 2008), CFA injection (Ji et al., 2002), capsaicin injection (Firner et al., 2006), chronic constriction injury (Tseng et al., 2007), partial sciatic nerve ligation (Kiguchi et al., 2009), and rhizotomy (Cheng et al., 2003). ERK has been shown to recruit AMPA to the membrane (Galan et al., 2004) allowing for more glutamate binding (Galan et al., 2004). ERK also phosphorylates the NR1 subunit of NMDA receptors increasing NMDA function (Slack et al., 2004; Kohno et al., 2008), and decreases potassium currents thereby making the cell more excitable (Hu et al., 2006, 2007). The major way for ERK to be activated is via calcium influx and adenylate cyclase activity (Wei et al., 2006). It is also important to note that ERK is a better marker for central sensitization than c-fos because ERK will only be activated in naïve conditions by intense noxious stimuli, whereas c-fos can be activated by innocuous stimulation (Hunt et al., 1987). Inhibition of ERK has been shown to decrease behavioral measures of central sensitization (Kawasaki et al., 2008). Importantly, partial sciatic nerve ligation has been shown to result in a significant down regulation in PER1 in dorsal horn neurons, due to transcriptional alterations (Morioka et al., 2016a).

Transcriptional changes can cause central sensitization to have serious staying power. ERK carries out its transcriptional actions through CREB and leads to expression of c-fos, NK1, TrkB, and cyclooxygenase-2 (COX-2) (Ji et al., 2009). Phosphorylated CREB exhibits a rhythm in the dorsal horn following chronic constriction injury as well as in the SCN, however, these rhythms differ (Xia et al., 2016), potentially due to the role CREB plays in the SCN as the level of pCREB increased at ZT10 (2 h before onset of the active phase). The ERK pathway is a great example of a signal’s actions leading to more of that signal. This positive feedback is what maintains chronic pain and may also lead to similar alterations along the pathway that relays pain-related information to the hypothalamus mentioned above, thus potentially leading to disruption of both peripheral and central clock genes as well as global behavioral circadian disruption (Figure 1).

### Disinhibition

Loss of inhibitory tone and disinhibition in the dorsal horn is one of the main drivers in triggering and maintaining central sensitization (Latremoliere and Woolf, 2009). Peripheral nerve injury can dysregulate the potassium-chloride co-transporter (KCC2) and lead to disinhibition. In extreme cases a normally inhibitory GABA signal can become excitatory. This is largely due to BDNF released by microglia (Coull et al., 2003), which will be discussed in more detail below, and restoring KCC2 returns spinal nociceptors to their normal state (Lavertu et al., 2014). Restoration of inhibitory controls has been implicated as a potential treatment mechanism for chronic pain by facilitating fast inhibitory neurotransmission and increasing norepinephrine inhibition (Zeilhofer et al., 2012; Hayashida and Obata, 2019). Immune cells and mediators can also lead to disinhibition in the central nervous system (Latremoliere, 2016), notably by T cells and microglia (Watkins et al., 2007; Old et al., 2016). These cells release mediators that play an active role in central sensitization, including IL-1β and IL-6 which inhibit the frequency of inhibitory post synaptic currents (IPSC’s) in lamina 2 neurons, and IL-1β which also decreases the amplitude of IPSC’s on the post synaptic cell (Kawasaki et al., 2008). TNF-alpha and IL-1β can both enhance excitatory signal and reduce inhibitory signal (Samad et al., 2001; Kawasaki et al., 2008). In addition, CCL2, has been shown to inhibit GABA currents in spinal neurons (Gosselin et al., 2005). Importantly, CCL2 and IL-6 production have been shown to be under the control of *Per1*, because when *Per1* is inhibited these cytokine levels are increased and when PER1 protein is overexpressed CCL2 and Il-6 levels are attenuated (Sugimoto et al., 2014). It has also been shown that TNF-α can suppress action potentials in GAD67+ cells and increase NMDA activity through phosphorylating ERK (Xu et al., 2010; Zhang et al., 2010), however, it should also be noted that not all cells expressing GAD in the CNS are inhibitory, as some of them express Vglut2 instead (Moffitt et al., 2018; Machado et al., 2020).

The disinhibition of spinal projection neurons, especially when paired with increased excitability, can expand their receptive field, which has been shown in animal models and humans (Woolf, 1983; Neziri et al., 2010). The expansion of receptor fields is a CNS process and not due to diffusion of inflammatory mediators, because the phenotype exists after blood flow restriction (LaMotte et al., 1991). Under normal conditions lamina 1 projection neurons have silent inputs from Aβ fibers from outside their receptive fields (Torsney and MacDermott, 2006). After peripheral inflammation, Aβ synaptic input to the dorsal horn is increased (Baba et al., 1999), and when the periphery of large A fibers are exposed to inflammatory factors they begin to express of BDNF and substance P (Mannion et al., 1996; Neumann et al., 1996). Additionally, after administration of either GABA or glycine antagonists, Aβ fibers are recruited to the superficial dorsal horn (Baba et al., 2003). In pathological condition these inputs can elicit a response, and in doing so a non-painful stimulus is perceived as painful (Latremoliere and Woolf, 2009; Latremoliere, 2016). Additionally, BDNF signaling through TrkB has been shown to activate pERK and lead to an increase in the clock genes BMAL1, CLOCK, and PER1, as well as *Per1* mRNA in spinal neurons after spinal cord injury. This effect was also increased in constant darkness due to an increase in melatonin signaling (Rea and Figueiro, 2013). Melatonin is a marker for circadian phase that is found to be at its’ highest expression in the middle of the night and is inhibited by light. Indeed, the disinhibition of spinal neurons seems to be a circadian-mediated process at the transcriptional level, given that circadian genes mediate CCL2 and TNF-α levels (Xu et al., 2010; Zhang et al., 2010; Sugimoto et al., 2014), and that can be altered by different environmental lighting paradigms.

While generally thought of as a spinal phenomenon, central sensitization has been shown in the brain, as well, and particularly in the thalamus (Dostrovsky and Guilbaud, 1990), the amygdala (Neugebauer and Li, 2003; Neugebauer et al., 2003), and the aforementioned LPB in the midbrain (Moylan Governo et al., 2006). The LPB is heavily innervated by pain projection neurons (Todd et al., 2000), and is home to an incredibly diverse neuronal population with roles in numerous physiological functions. Most neurons in lamina 1 of the lumbar enlargement follow the anterior-lateral tract and can be retrogradely labeled from the LPB, about 90% of which express NK1R (Cameron et al., 2015). Thus, the connection between afferents expressing substance P, NK1R spinal neurons, and the LPB seems to be crucial for rhythmicity of pain. Tac1, which codes for substance P, was found to be rhythmically expressed in the DRG under the control of the BMAL1/CLOCK heterodimer and the amount of substance P in the dorsal horn followed this same rhythm, as did the rhythm of hypersensitivity in response to formalin. Additionally, when NK1 receptors are blocked the rhythmicity of pain intensity in response to formalin injection was lost (Zhang J. et al., 2012). These NK1R projection neurons have been shown to be functionally connected to the pdyn neurons in the dorsal LPB (dlPB). From there these cells seem to mediate a plethora of pain-related effects including escape behaviors, analgesia, and aversive memory (Chiang et al., 2020).

Neuropathic pain and capsaicin pain both can be attenuated following either optogenetic or chemogenetic inhibition (Chiang et al., 2020; Sun et al., 2020). Additionally, driving LPB glutamatergic neurons leads to a neuropathic pain phenotype without the need of injury, but in the case of injury, such neuropathic pain can be halted by stimulating GABAergic neurons in the LPB. Importantly, when these cells are stimulated once a day for 7 days their effects become chronic, such that persistent stimulation of LPB glutamatergic neurons leads to neuropathic pain that is sustained for at least a month after any exogenous stimulation and this can be transiently inhibited by optogenetic stimulation of LPB GABAergic neurons. Additionally, when GABAergic neurons in the LPB are stimulated for the first 7 days of common peroneal nerve ligation, they can completely stop the development of neuropathic pain (Sun et al., 2020). This is achieved through a monosynaptic connection of LPB inhibitory neurons onto LPB excitatory neurons. These findings show that the LPB can exhibit a gain in excitability and disinhibition, the hallmarks of central sensitization, and that increasing its inhibitory tone relieves pain. Projections from the dlPB to the VMH and PAG mediate escape behaviors, while the dlPB to PAG circuit also mediates analgesia through the endogenous opioid system (Chiang et al., 2020). Additionally, pdyn cells in the dlPB that project to the external lateral PB (elPB) mediate aversion through connections to the bed nucleus of the stria terminalis (BNST) and central amygdala (CEA). Given the connections between the PB and the circadian system that involve glutamatergic and pdyn PB cells discussed above, it seems likely that any central sensitization found in these cells could very well influence the circadian system.

**FIGURE 3:**
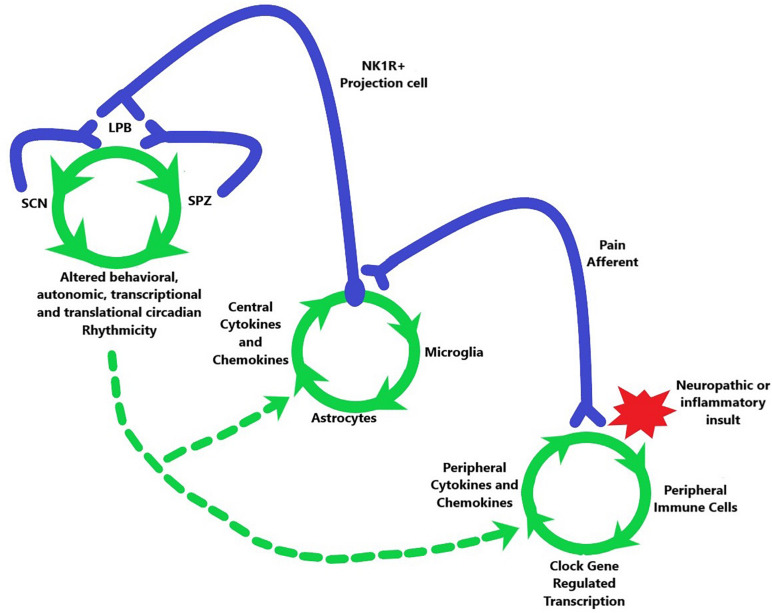
Potential model for the travel of the pain induced positive feedback system, initiated by a neuropathic or inflammatory insult (red star), from the periphery (lower right green circle) to the spinal cord (middle green circle) and then to the brain (upper left green circle). Green arrows indicate order of activation or alteration in each system. This travel leads to altered circadian output of multiple physiological systems (where green arrows converge at bottom of top left green circle). The circadian system is also able to affect the pathway through axonal connections back from the SPZ and SCN to the LPB as well as by modulating the spinal and peripheral phenomena in chronic pain. This modulation has differing effects depending on the circadian time which is indicated by dashed green arrows.

Taken together there appears to be a circuit connecting peripheral activation to central sensitization within CNS nuclei that mediate circadian rhythms (Figure 3). Peripheral pain afferents become activated by inflammatory or neuropathic insults that lead to a rhythmically mediated substance P release onto NK1R-expressing projection cells, which then transmit this signal to the LPB including a functional connection to LPB pdyn cells (Chiang et al., 2020). The LPB then appears to send information to both the SCN^VIP^ cells and to the SPZ, which in turn project back to the LPB. The neurons within each of these stops are subject to circadian-mediated gain in excitation and disinhibition, which could be transmitted along the entire circuit. Indeed, central sensitization as seen in the spinal cord, following nerve ligation, has been shown in the LPB to be able to drive both physical and affective components of pain (Sun et al., 2020). Importantly, the maintenance of chronicity is also mediated by glial cells that contribute heavily to the neuroinflammation that maintains pain long after any potential tissue damage.

## Neuroinflammation

Pain is transmitted from the periphery to the CNS through neural connections, but these neural connections are not the whole story (Figure 2). Interaction with immune cells, activation of glial cells, and production of inflammatory mediators characterizes neuroinflammation (Ji et al., 2014, 2016). Importantly, these actions often take place through intracellular mitogen activated protein kinase (MAPK) pathways including JNK, ERK, and p-38 (Ji et al., 2009). In this section we will focus on central neuroinflammation, however, it is important to recognize that any sensitization of the periphery will aid in central induction of inflammation (Latremoliere and Woolf, 2009; Scheiermann et al., 2013; Ji et al., 2014; Segal et al., 2018; Donnelly et al., 2020).

Central sensitization of the spinal cord involves activation of astrocytes, microglia and T cells (Raghavendra et al., 2004; Gao and Ji, 2008; Ellis and Bennett, 2013; Ji et al., 2014; Woller et al., 2017). These glial cells release proinflammatory factors in the central nervous system like PGE-2, NO (Matsui et al., 2010), IL-1β, IL-8, TNF-α, IL-6 (Woller et al., 2017), as well as CXCL1/2 (Zhang et al., 2013), and CCL2 (Gao et al., 2009b). The activation of microglia and astrocytes are associated with both inflammatory and neuropathic pain states. Raghavendra et al. (2004) found that CFA injections induced microglial activation, but not astrocytic activation, in the induction phase of CFA inflammation (4 h), followed by activation of both types of glial cells in the persistent phase (day 4 and day 14), as well as increased TNF-α, IL-1β, and IL-6 at all three times points. Importantly, these changes were measured at L5, the brainstem, and the forebrain. This implies a systemic central inflammatory issue that is not locked in place at the spinal cord level but can travel to various nuclei in the CNS.

Glial mediators, whether large (cytokines, chemokines, growth, and inflammatory factors) or small (Glutamate, ATP, D-serine) can effect neuronal and synaptic activity at nanomolar concentrations (Ji et al., 2013). These mediators have overlapping functions and origins, and each have been associated with bidirectional interactions with the circadian system. We will focus on them in the context of microglia and of astrocytes.

### Microglia

Microglia are the resident immune cells of the CNS and as such they are prime targets to examine the interplay of immune, circadian and pain signaling (Burke et al., 2016; Inoue and Tsuda, 2018). Microglia are the major source of TNF-α in the spinal cord and seem to mediate the onset of chronic pain through chemokine and cytokine interactions, including CX3CL1/CX3CR1 signaling (Ji et al., 2014). In addition to their established role in the spinal cord (Inoue and Tsuda, 2018), microglia also play a role in the brain. For instance, microglia have been shown in the amygdala to relate to anxiety associated with pain (Sawada et al., 2014), in the mesolimbic system to disrupt reward behavior (Taylor et al., 2015), and in hippocampal synaptic plasticity (Liu et al., 2017). Microglia also play a role in synaptic transmission in healthy adult brains (Tremblay et al., 2011), and in the developing brain (Weinhard et al., 2018).

Microglia appear to be mostly involved in the initiation phase of chronic pain (Ji et al., 2013, 2014, 2016; Segal et al., 2018; Donnelly et al., 2020). Indeed, after nerve injury microglia undergo rapid proliferation that is observed within 2 days (Suter et al., 2007). Additionally, low frequency stimulation of rat c-fibers can activate spinal microglia independent of painful pre-conditioning (Hathway et al., 2009). However, blocking c-fibers after spared nerve injury was not enough to block spinal microglial activation (Suter et al., 2009), while interrupting microglial proliferation does attenuate hypersensitivity (Gu et al., 2016). This implies that, once neuroinflammation is activated, stopping nociception does not stop pain. The intracellular mechanisms mediating microglial activity are also crucial for the development of pain hypersensitivity, and inhibiting this activation decreases pain behaviors (Raghavendra et al., 2003; Grace et al., 2016, 2018). Importantly, the majority of work involving microglia has been done in males, and in females there is a severely limited attenuation of pain when microglia are inhibited. In fact, it seems that females can use T-cells in place of microglia to drive chronic pain (Sorge et al., 2015). Additionally, there is evidence of an entirely distinct pathway of nociception and inflammatory pain in females that is mediated by the melanocortin-1 (MC1) receptor (Delaney et al., 2010).

Microglial-mediated inflammation in response to LPS follows a pattern with the worst inflammatory response occurring during the light phase (Dantzer et al., 2008). Fonken et al. (2015) showed that microglial cytokine response to LPS was worst during the light phase and that microglia harvested during the light phase and treated with corticosterone induces expression of Per1 mRNA. However, microglia from adrenalectomized mice still show rhythmic expression of both circadian and cytokine mRNA. This implicates corticosterone as an influencer of circadian cytokine expression but not the driver of these rhythms. This is similar to the influence that hormones can have on neuronal rhythms without actually being the main driver of entrainment.

Phosphorylation of the MAPK p38 is a major marker for microglial activation after spinal cord injury (Hains and Waxman, 2006; Crown et al., 2008), chronic opioid exposure (Cui et al., 2006; Watkins et al., 2009), nerve injury (Jin et al., 2003; Kobayashi et al., 2008), post-operative pain (Wen et al., 2009; Peters and Eisenach, 2010), and formalin injection (Svensson et al., 2003), among many others. Importantly, proinflammatory mediators and neurotransmitters that have been associated with chronic pain, such as CX3CR1 which is only expressed on microglia (Zhuang et al., 2007), CCL2 (Cho and Gruol, 2008; Thacker et al., 2009), ATP (Gong et al., 2009), TNF-α (Svensson et al., 2005), IL-1β (Sung et al., 2005), and CGRP (Wang Z. et al., 2010), also lead to phosphorylation of p38. This shows the importance of phosphorylated p38 in chronic pain. However, this too is sexually dimorphic, as spinal inhibition of p-p38 is effective in treating pain in male mice but not in female mice (Taves et al., 2016). Most notably the activation of microglia and the phosphorylation of p38 is important because it leads to transcriptional changes and upregulations in genes that code for the very mediators that activated the MAPK pathway to begin with. These include TNF-α, IL-1β, and BDNF (Wen et al., 2011). TNF-α specifically alters transcription of circadian genes including *Per1/2* and BMAL1 (Hashiramoto et al., 2010). In this same study it was found that *Cry1/2* deleted mice showed worse arthritic scores with elevated TNF-α, IL-1β, and IL-6. The increased arthritic score in *Cry*-deleted mice was dampened by intraperitoneal anti-TNF-α antibody administration. Additionally, spinal injection of microglia that have been activated with ATP produces mechanical allodynia via BDNF release in males, and this is attenuated by BDNF blockade (Coull et al., 2005). However, in females ATP-activated microglia did not induce mechanical allodynia, nor did intrathecal BDNF inhibition attenuate pain (Mapplebeck et al., 2017, 2018). However, Sikandar et al. (2018) found that BDNF deletion from only sensory afferents led to reduced chronicity in one model of neuropathic pain but not another, while holding true for both female and male mice. This implies a difference in BDNF’s effects depending on its origin as well as the sex of the mouse. Nevertheless, this positive feedback is a common theme in numerous types of inflammatory and neuropathic pain (Gao and Ji, 2008).

Purinergic receptors like P2X4, P2Y12 (Kobayashi et al., 2008; Trang et al., 2009), and numerous other subtypes like P2X7 (Ji et al., 2013), as well as Toll-like receptors (TLR’s) such as TLR2, TLR4, and TLR9 (Nicotra et al., 2012), also play a role in the cyclic nature of neuroinflammation (Tsuda et al., 2010; Trang et al., 2012; Inoue and Tsuda, 2018). Activation of P2X7 leads to IL-1β release (Clark et al., 2010), as does activation of TLR2 and TLR4 (Tanga et al., 2005; Kim et al., 2007). Additionally, CCL21 and CCL2 increase expression of P2X4 on microglia (Biber et al., 2011; Toyomitsu et al., 2012). Purinergic receptors have also been implicated in opioid tolerance (Horvath and DeLeo, 2009; Ji, 2010), although this is debated (Ferrini et al., 2013). Microglia release IL-1β (Hanisch, 2002; Mousseau et al., 2018; Donnelly et al., 2020), and this increases NMDA and AMPA neurotransmission, while decreasing glycine and GABA neurotransmission (Kawasaki et al., 2008). Furthermore, nerve ligation upregulates BDNF in spinal microglia via P2X4 (Ulmann et al., 2008; Trang et al., 2012), while activation of P2X4 resulted in BDNF expression by phosphorylating p38 (Trang et al., 2009).

Pain behaviors are severely attenuated in mice that are genetically deficient in, or have a pharmacologic block on, many of the microglial mediators mentioned above. Genetic deletion of CCR2 attenuates neuropathic pain (Abbadie et al., 2009). TLR2 knockout mice experience reduced neuropathic pain after nerve injury (Kim et al., 2007), as do TLR4 mutant mice (Tanga et al., 2005), however, this effect was again restricted to males (Mogil, 2012). P2X4 deleted mice have blunted responses to both inflammatory and neuropathic pain phenotypes (Tsuda et al., 2009). Microglial reaction that was induced by nerve injury was abolished in CCR2 knockout mice (Zhang et al., 2007), and neuropathic pain is lessened by a CCR2 antagonist (Zhang Z.J. et al., 2012). It is important to point out, however, that minocycline, a selective microglial inhibitor, is effective in animals but has not been in humans (Vanelderen et al., 2015; Sumitani et al., 2016; Curtin et al., 2017).

Interestingly, recent work has shown that using diphtheria (DT) toxin to selectively delete microglia expressing the DT receptor in the SCN leads to an increase of Per1 mRNA and a decrease in BMAL1 after 48 h. Additionally, body temperature rhythms stayed elevated for 3 days after DT while energy expenditure and activity stayed reduced (Sominsky et al., 2021). IL-1β and NF-κB activation via inflammasome action, which will be discussed below, are inhibited by NPY action on microglia (Ferreira et al., 2010). NPY released from the IGL onto SCN^VIP^ cells also appears to mediate the entrainment of circadian rhythms induced by time-dependent access to a running wheel (Janik et al., 1994), a model of voluntary exercise-induced entrainment. Together this implies the possibility of a mechanism for the exercise-induced alterations of rhythms and anti-inflammatory actions of NPY to be synchronized in the SCN and for this to then lead to improvement of various painful pathologies through exercise. Indeed, inflammatory pain in RA has been shown to be improved through exercise (Kavuncu and Evcik, 2004).

### Astrocytes

Astrocytes are heavily implicated in pain behaviors and their activation may be even more associated with chronic pain than microglia (Colburn et al., 1997). In fact, they appear to stay activated for the full length of the chronic pain (Zhang and De Koninck, 2006; Ji et al., 2013). Furthermore, the effect of astrocytes in chronic pain seems to be largely conserved in both males and females (Chen et al., 2018; Ji et al., 2019; Donnelly et al., 2020). Release of small molecules like D-serine, an NR1 glycine site agonist (Nong et al., 2003; Miraucourt et al., 2011), ATP and glutamate from astrocytes enhances pain pathology (Gao and Ji, 2010; Halassa and Haydon, 2010). Astrocytes are deeply interconnected with both synapses and blood vessels, and this positioning allows them to mediate the blood–brain barrier (Halassa and Haydon, 2010). In fact, the BOLD (blood oxygen level dependent) signals on an fMRI are mediated by astrocytes (Iadecola and Nedergaard, 2007). Astrocytic activation is seen primarily through hypertrophy, generally as increased GFAP labeling, or induction of phosphorylated JNK (Ji et al., 2014). Astrocytic activation through pJNK has been extensively linked to neuropathic pain conditions (Zhuang et al., 2006), inflammatory pain conditions (Gao et al., 2010), skin cancer pain (Gao et al., 2009a), intrathecal bFGF (Ji et al., 2006), bone cancer (Wang et al., 2012), and TNF-α challenge (Gao et al., 2009b). It has also been shown that neuronal activity drives astrocyte activation and that blocking pain afferents reduces astrocyte activation (Xie et al., 2009; Wang H. et al., 2010). Nam et al. (2016) showed that, using channelrhodopsin-assisted circuit mapping (CRACM), activating astrocytes leads to pain hypersensitivity, release of ATP, NR1 phosphorylation, and increased mRNA for TNF-α, IL-1β, IL-6, and CCL2. This was also shown to reduce the frequency of action potentials in GABA neurons and to activate NK1R projection neurons, as shown with c-fos (Nam et al., 2016). General inhibition of astrocytes has shown effectiveness in reducing pain behaviors (Zhuang et al., 2006; Chen et al., 2012). Given these factors, and the roles that astrocytes play in circadian rhythms (see below), understanding astrocytes in depth is crucial.

Inhibiting JNK activation has been shown to attenuate chronic pain, and D-JNKI-1, a selective JNK1 inhibitor, has been shown to attenuate pain measures (Zhuang et al., 2006; Gao and Ji, 2008; Gao et al., 2010). SP600125, a more general JNK inhibitor, has also shown effectiveness (Morioka et al., 2016a). Since the primary pathway to persistent pain mediated by MAPKs is to alter transcription (Gao and Ji, 2008), it makes sense that blocking these signaling molecules is effective. However, the efficacy of them is limited by their time course. Usually, the reduction in pain has a limited time frame (Zhuang et al., 2006). Furthermore, these MAPKs are largely ubiquitous in cells throughout the body so, long term inhibition of them carries tremendous risk.

Importantly, astrocytes have also long been known to exhibit circadian rhythms and play a role in circadian function. GFAP is expressed rhythmically in the SCN (Lavialle and Serviere, 1993; Santos et al., 2005), and cortical astrocytes have been shown to have their own intrinsic circadian rhythmicity of *Per* genes tagged with luciferase (Prolo et al., 2005). Astrocytic rhythms interact with androgens, as shown by increased GFAP in castrated male mice along with decreased synaptophysin and dysregulation of *Per* genes in the SCN (Karatsoreos et al., 2011). More recently, astrocytes have come to be characterized as being equal partners in synchronizing circadian rhythms within the SCN (Brancaccio et al., 2017, 2019). These experiments have shown not only that astrocytes in the SCN have their own TTFL’s, but also that these cells can influence behavioral rhythms through glutamate signaling with neurons. SCN neurons are active during the day and are hyperpolarized at night, whereas astrocytes operate on an opposite schedule (Hastings et al., 2018). Glutamate released from astrocytes is thus high at night in the SCN and can inhibit neuronal activity through binding to the NR2C subunit of NMDA receptors that are expressed on the presynaptic terminals of neurons projecting to the dorsal SCN (Brancaccio et al., 2017), leading to GABA release and therefore an increase in inhibitory action within the SCN. Furthermore, blockade of EAAT3, the glutamate transporter expressed on astrocytes, is known to disrupt SCN rhythms (Cagampang et al., 1996). Additionally, under normal conditions, EAAT3 is rhythmically expressed in the SCN where it is highest in the late dark phase and into the early light phase, leading to an uptake of extracellular glutamate and a lessening of the NR2C mediated GABAergic inhibition onto SCN dorsal neurons. TNF-α administration to astrocyte cultures has been shown to alter both phase and amplitude of PER2 rhythms at CT13/14, but not CT1 (Duhart et al., 2013). Media from astrocytes challenged with TNF-α, or LPS, also induced a phase shift in neuronal *Per1* rhythms, and intracerebroventricular injection of TNF-α *in vivo* also phase delayed LMA rhythms. Altogether, these data support the conclusion that SCN astrocytes are active members in circadian physiology.

Interestingly, TNF-α treatment onto astrocytes increases CCL2 mRNA, but this is attenuated with application of a REV-ERBα agonist (Morioka et al., 2016b). Furthermore, REV-ERBα treatment can inhibit expression of CCL2 and CX3CR1 in macrophages, which can infiltrate the CNS in pathological pain condition (Lam et al., 2013; Sato et al., 2014). More recently, the mechanism of this REV-ERBα mediated decrease in inflammatory factors was shown to be enacted through inhibiting NF-κB translocation to the nucleus and blocking transcription of its target genes within microglia. It was even shown that neurons were protected from microglia by REV-ERBα agonist administration into the ventral midbrain (Guo et al., 2019; Pourcet and Duez, 2020). Given the role of REV-ERBα in the accessory loop that stabilizes the TTFL, its role in regulating inflammatory and pain responses represents another link between the mediators underlying pain and circadian rhythmicity that warrants further study. Furthermore, partial sciatic nerve ligation has been shown to decrease *Per1*, *Cry1* and PER1 levels in astrocytes and neurons of the spinal cord at onset of the dark phase 7 days post-surgery in mice. This result was copied with intrathecal administration of *Per1* siRNA which also increased pJNK levels along with decreasing paw withdrawal threshold which was rescued by inhibiting pJNK. *Per1* siRNA also increased CCL2 levels and induced a rhythmicity in CCL2 content that peaked at ZT18. This siRNA-induced mechanical hypersensitivity was also blocked by a CCL2 antagonist (Morioka et al., 2016a). These results taken together argue that balance of the circadian system is severely disturbed in chronic pain, as well as being self sustaining through a glial mechanism. Rectification of the circadian system may present an effective way to treat these issues.

## Discussion and Future Directions

As we have reviewed here, there are overlapping mechanisms underlying circadian rhythmicity and pain-related processes such as peripheral activation, central sensitization, and neuroinflammation driven by neurons, microglia, and astrocytes. There is also shared circuitry, especially involving the LPB, between descending circadian output and ascending pain-related pathways, which may influence higher-order interactions of perceptual and affective responses as well as endogenous analgesia. Circadian-related treatments have already shown promise in treating pain, and applying the information reviewed here presents many opportunities for greater clinical impact on chronic pain pathologies and better maintenance of circadian health in such conditions. Numerous medications already in clinical use, such as non-steroidal anti-inflammatory drugs (NSAIDs), cytokines, and glucocorticoids, have been found to be more effective in treating a wide range of conditions, including RA, if given at the proper circadian timepoint (Walton et al., 2020).

Melatonin, which is regulated by the SCN via a multisynaptic pathway, which provides one of the most reliable markers of internal circadian phase in humans by its nocturnal secretion, can be used therapeutically to modulate pain and inflammation. Melatonin has been shown to reduce both hyperalgesia and allodynia in neuropathic, acute, and inflammatory pain (Srinivasan et al., 2010), and has been shown to inhibit an LPS-induced increase in TNF-α and lead to increased levels of the anti-inflammatory IL-10 (Carrillo-Vico et al., 2005). Importantly, melatonin also has reciprocal actions on the SCN, which heavily expresses both major types of melatonin receptor (Dubocovich, 2007). Additionally, melatonin suppresses IL-8 in lung fibroblasts through an ERK cascade (Kim et al., 2012), and blocks the activation of microglia by methamphetamine as well as the cardiac release of inflammatory cytokines following exercise (Veneroso et al., 2009; Tocharus et al., 2010). This block of inflammation after exercise is carried out through the modulation of NF-κB in skeletal muscles as well (Alonso et al., 2006). This shows that melatonin can act upon microglia and impact exercise-driven inflammation, and further raises the possibility of interactions between pain, the circadian system, and exercise, as a circuit substrate for exercise-induced entrainment of the SCN has already been suggested through NPY signaling from the IGL via the GHT (and, as mentioned previously, NPY also appears to have its own anti-inflammatory actions through the inhibition of microglia). Additionally, intracellular MAPK cascades are altered by melatonin, however, its changes to JNK, p38, or ERK levels appear to be receptor dependent (Mauriz et al., 2013).

Melatonin’s anti-inflammatory actions are partially mediated through NF-κB modulation (Mauriz et al., 2013). This means that melatonin can modulate the NOD-like receptor protein 3 (NLRP3) inflammasome, sometimes called NALP3 (He et al., 2016), which underlies the regulation of proinflammatory cytokines (Cuesta et al., 2010). Indeed, neuroinflammation and intracellular peripheral inflammation are largely mediated by such inflammasomes, which can be found within astrocytes, microglia and neurons in the CNS (Chen et al., 2018; Donnelly et al., 2020). NLRP3 is crucial to the maturation and secretion of IL-1β and IL-18 (Cowie et al., 2019; Chan and Schroder, 2020; Pourcet and Duez, 2020), and as such, NLRP3 activation is associated with neuropathic (Ji et al., 2021; Jia et al., 2017), postoperative (Cowie et al., 2019), neurodegenerative (Ahmed et al., 2021; de Araujo et al., 2021), and inflammatory injuries and diseases (Shao et al., 2015; Chen et al., 2018; Pourcet and Duez, 2020). NLRP3 activation can be achieved through many of the changes that are common in neuroinflammation and central sensitization, including potassium efflux (Kelley et al., 2019), binding of ATP to purinergic receptors (Silverman et al., 2009; Di Virgilio et al., 2017), TLR activation (Song et al., 2017; Inoue and Tsuda, 2018), as well as either TNF-α or IL-1β signaling (Yazdi and Ghoreschi, 2016; Pourcet and Duez, 2020). Upon such activation, JNK has been shown to mediate phosphorylation and subsequent activation of NLRP3 (Song et al., 2017), giving credence to the JNK activation seen especially in astrocytes during chronic pain. In fact, when JNK is inhibited, NLRP3 activation is reduced (Song et al., 2017). Upon NLRP3 activation the NF-κB pathway is activated, which interacts with the circadian system (Marpegan et al., 2004; Spengler et al., 2012), and leads to transcription of the interleukin mRNA as well as NLRP3 mRNA (Pourcet and Duez, 2020). This framework for creating and maintaining inflammation is under the control of the circadian system and has shown potential to be treated by circadian intervention. NLRP3 inflammasome expression is highest during the dark/active phase in rodents due to its transcriptional controller REV-ERBα being lowest (Pourcet et al., 2018; Cornut et al., 2020), which is the first line of defense against inflammation. As such, when REV-ERBα levels are disturbed, Il-1β levels increase (Yu et al., 2019). REV-ERBα ablation alone leads to activation of NLRP3 in mice because REV-ERBα represses transcription of the inflammasome as well as exercising a secondary inhibition through the NF-κB pathway. REV-ERBα activation by pharmacologic intervention was also shown to attenuate experimental inflammatory colitis (Wang S. et al., 2018). This implies a potential of treatment for inflammatory conditions by directly stimulating REV-ERBα of the circadian system.

TNF-α, IL-1β, and IL-6 peak in the blood around the beginning of the active phase in both mice and humans (Haus et al., 1983; Haus and Smolensky, 1999; Cutolo, 2012; Scheiermann et al., 2013). During this time, mice are more sensitive to inflammatory cytokines (Hrushesky et al., 1994; Scheiermann et al., 2013), and humans with rheumatoid arthritis experience stiffness and pain (Cutolo, 2012). In line with these rhythms, stimulating TLR4 during the start of the active phase produced increased levels of IL-6, CCL5, CXCL1, and CCL2 as compared to stimulation at the start of the resting phase. Genetic deletion of REV-ERBα or macrophage deletion of Bmal1 ablated this time-dependent effect (Gibbs et al., 2012). Interestingly, TLR4 does not present with a circadian rhythm, however, its intracellular messengers do (Keller et al., 2009; Labrecque and Cermakian, 2015). Rhythms of CCL2 have been found in basal conditions as well as under LPS, a TLR4 ligand, challenge (Hayashi et al., 2007; Gibbs et al., 2012; Nguyen et al., 2013; Scheiermann et al., 2013). Activation of the NF-κB pathway and IL-1β by a TLR5 agonist varies rhythmically with time of day (Spengler et al., 2012). Many of these studies were done in macrophages or leukocytes. However, during neuroinflammation these cells infiltrate the CNS. There is even evidence that leukocyte infiltration to tissues could exhibit its own rhythm (Scheiermann et al., 2013). Furthermore, mice with experimental arthritis showed a significantly decreased arthritis score when given baricitinib, a cytokine blocker, at ZT0 rather than ZT12 (Yaekura et al., 2020). This again highlights the temporal dependency of chronic pain at the behavioral and treatment level.

Drugs that affect catecholamines, which are also key mediators of the stress response and of pain control (Kambur and Mannisto, 2010), also hold promise for treating chronic pain in a chronotherapeutic fashion. Humans and mice have high circulating catecholamine levels during the active phase (Linsell et al., 1985). For example, epinephrine shows an acrophase in the blood at the start of the active phase, similar to TNF-α and IL-1β (Haus et al., 1983; Haus and Smolensky, 1999; Scheiermann et al., 2013). Norepinephrine does not show a circadian rhythm in blood but does show rhythmicity infiltrating tissues, which influences melatonin secretion (Leach and Suzuki, 2020). These levels of catecholamines are regulated by the hypothalamic pituitary adrenal axis (HPA), and it has been shown that clock genes within adrenal nerves respond to light-induced phase shifts (Ishida et al., 2005). However, these responses are lost upon SCN lesion (Niijima et al., 1993), implying a circuit by which the SCN modulates the adrenal gland. Importantly, serotonin and norepinephrine reuptake inhibitors (SNRI’s) have shown some efficacy in treating neuropathic pain. These seem to largely depend on the α-adrenergic receptors (α-AR) through increased inhibitory input from cholinergic cells within the spinal cord and hyperpolarization due to influx of potassium (Obata, 2017). Intraperitoneal administration of the SNRI duloxetine reduces mechanical hypersensitivity for up to 24 h but eventually disappears (Ito et al., 2018). Studies like these indicate the potential to treat pain by inhibiting reuptake of norepinephrine, and indeed these SNRI’s have shown some efficacy in clinical medicine (Finnerup et al., 2015).

Alternatively, there have also been studies indicating that sustained spinal and systemic norepinephrine can lead to an increased pain phenotype (Hartung et al., 2014; Zhang et al., 2018; Sugama et al., 2019). These studies were carried out over a longer time course and found that sustained inhibition of norepinephrine breakdown led to increased pain response for up to 3 weeks after stopping the inhibition and relied on β adrenergic receptors (β-AR). These studies implicated glial cells driving neuroinflammation as the primary cause of this sustained increase in pain. In line with this, there were elevated levels of TNF-α, IL-1β, IL-6, and CCL2 in the spinal cord as well as increased p-p38 and pERK signaling (Hartung et al., 2014; Zhang et al., 2018). Additionally, in humans, the genetic variant that codes for low levels of catechol-*O*-methyltransferase (COMT), which breaks down norepinephrine, is associated with increased pain following capsaicin in women but not men (Belfer et al., 2013). While another form of the COMT gene is associated with high stress in men but not women (Meloto et al., 2016). Taken together, these data suggest a short-term role for norepinephrine in reducing neuropathic pain but a role in sustained pain should norepinephrine stay elevated in the spinal cord too long, as well as a possible sex difference that is not yet explained mechanistically. Importantly, norepinephrine signaling interacts with circadian genes. Norepinephrine treatment onto cultured spinal astrocytes increased *Per1* mRNA, and this could be blocked by antagonizing ERK, JNK, PKA, α-AR, or β-AR. Blocking *Per1* upregulation was most effective upon blockade of either both ARs or blockade of all three kinases (Sugimoto et al., 2011). Beta blockers have also shown some efficacy in managing mechanical allodynia in mice with both neuropathic and inflammatory pain (Martin et al., 2015). In the future more research will be needed to elucidate the mechanisms of norepinephrine pain generating and pain repressing actions and how this is related to the daily rhythms of endogenous norepinephrine.

As mentioned above, bright light therapy has also shown some efficacy in treating patients with chronic pain. Both blue and green light have been shown to decrease paw withdrawal latency, a measure of hyperalgesia in WT rats (Khanna et al., 2019). Green light has been most heavily investigated for clinical use. In a recent study, it was shown that green light therapy, which activates the circadian system but to a lesser extent than blue light (Lockley et al., 2003), was able to improve multiple self-reported measures of pain and quality of life in mostly female fibromyalgia patients (Martin et al., 2021). Patients reported significantly lower overall numeric pain scores, and significantly lower associational scores of their pain with a number of descriptors such as, stabbing, aching, sharp, throbbing, shooting, tender, and tiring. Additionally, these patients reported having a much easier time falling asleep, staying asleep and exercising, which are all heavily regulated by the circadian system. The study participants were instructed to use the green LED lights at their own homes for between 1 and 2 h while in an otherwise dark room for 10 weeks. While this study did achieve good results, they did not examine differences between the male and female participants, nor did they report advising them to utilize this system at any particular time of day. This may have introduced a potential confounding variable that must be considered in future studies in humans and in animals.

A different study in rats showed that red light exposure for either 8 or 3 h a day led to hyperalgesia without the need for any physical injury or intentional inflammation (Khanna et al., 2019). This group reported that red light induced increasing hyperalgesia with either increased luminance or consecutive days of prolonged red-light exposure and that could be returned to baseline by reverting back to normal room light. Moreover, this increase in hyperalgesia and mechanical allodynia was measured at the paw and the shoulder, showing that this was a systemic hyperresponsiveness to mechanosensation and to pain that could be inhibited by blocking GABA-A receptors with bicuculline injected into the rostral ventral medial medulla (RVMM). The finding that prolonged green or blue light exposure, which stimulate the circadian system, can lead to analgesia while red light, which does not stimulate the circadian system (Enezi et al., 2011), leads to hyperalgesia is fascinating. However, this study did not specify what point in the circadian day the prolonged light exposure happened. This represents a potentially significant confound given the known circadian-dependent and wave-length-dependent effects of light on the SCN and on behavioral and physiological rhythms, meaning that these rats may have experienced phase shifts depending on what type of light they were exposed to and at what zeitgeber time. Additionally, the effect could indeed be largely circadian all together. If these mice were put into the prolonged red-light environment during their normal light period (which does not stimulate the circadian system), then they could essentially be experiencing circadian night for the entirety of their time in the red-light condition, while the prolonged green light exposure mice would be more correctly oriented to the circadian day. Additionally, prolonged red-light exposure has been shown to severely blunt the melatonin rhythm while also phase advancing the corticosterone rhythm in rats that are kept in a continual 12:12 light/red-light condition (Dauchy et al., 2015). Both rhythm disruptions can influence pain, though it is unclear how the red light exerts these influences. The influence of the circadian system on pain processing is not trivial, and as we review here appears to be quite impactful. Thus, circadian alterations must be considered when performing pain-related experiments that involve prolonged light alterations.

Moving forward we will need to look above the canopy to see how the interacting processes that underlie pain and circadian rhythms are linked. Pain is clearly not confined to the spinal cord, and we will thus need to understand how the sensitization and neuroinflammation that plagues the spinal cord is transmitted to higher brain structures, and how these signals affect different functions such as circadian rhythmicity. Likewise, we will need to better understand how the circadian system regulates pain processing both locally and globally and the impact on pain perception and efficacy of treatments. The overlap of signaling pathways, such as the MAPK’s, used and disrupted in pain processing as well as circadian function leaves the door open for dysfunction of one system to influence the other through the circuits that we have described here. Understanding such bidirectional interactions that influence pain signaling will be vital to finding ways in which we can better manage chronic pain. Importantly, we will also need to utilize models of both males and females at all stages of study, as the mechanisms that govern chronic pain and circadian function can differ greatly between sexes and these differences are incompletely understood largely due to the frequent bias of using male over female subjects in preclinical rodent work. It is unclear if such reported sex differences in circadian function and pain are due to common sex-linked traits or due to modulation by estrogen cycling in females. However, given the known association of estrogen in the circadian function of reactive astrocytes (Fernandez-Galaz et al., 1999; Lembach et al., 2018), such continual bias of male over female subjects is likely to miss some important interactions between circadian and pain-related processes and to continue to develop ineffective treatments for women. Our approach to finding these mechanisms needs to be nuanced, as we must never forget that every cell in our body that has DNA has both a genetic circadian clock and a genetic sex. Thus, in the hopes of one day finding ways to effectively treat chronic pain without causing more problems than we solve, we must recognize how these systems interact and how sexes differ. Most importantly, we cannot get lost in the weeds and the molecules only to forget that we are treating people, and that these people have different pathologies with different complications. A silver bullet may not be feasible, but treatment that uses the body and its endogenous rhythms to our advantage could prove to be a powerful tool.

## Author Contributions

AW and WT developed the idea for the manuscript. AW, JP, and WT wrote and edited the manuscript. All authors contributed to the article and approved the submitted version.

## Conflict of Interest

The authors declare that the research was conducted in the absence of any commercial or financial relationships that could be construed as a potential conflict of interest.
